# Degradation of Pesticide Residues in Water, Soil, and Food Products via Cold Plasma Technology

**DOI:** 10.3390/foods12244386

**Published:** 2023-12-06

**Authors:** Phanumas Sojithamporn, Komgrit Leksakul, Choncharoen Sawangrat, Nivit Charoenchai, Dheerawan Boonyawan

**Affiliations:** 1 Graduate Program in Industrial Engineering, Department of Industrial Engineering, Faculty of Engineering, Chiang Mai University, Chiang Mai 50200, Thailand; phanumas_sojit@cmu.ac.th; 2 Department of Industrial Engineering, Faculty of Engineering, Chiang Mai University, Chiang Mai 50200, Thailand; choncharoen@step.cmu.ac.th (C.S.); xnivit@gmail.com (N.C.); 3 Plasma and Beam Physics Research Center (PBP), Faculty of Science, Chiang Mai University, Chiang Mai 50200, Thailand; dheerawan.b@cmu.ac.th

**Keywords:** cold plasma technology, food safety, pesticide degradation, nonthermal technologies

## Abstract

Water, soil, and food products contain pesticide residues. These residues result from excessive pesticides use, motivated by the fact that agricultural productivity can be increased by the use of these pesticides. The accumulation of these residues in the body can cause health problems, leading to food safety concerns. Cold plasma technology has been successfully employed in various applications, such as seed germination, bacterial inactivation, wound disinfection, surface sterilization, and pesticide degradation. In recent years, researchers have increasingly explored the effectiveness of cold plasma technology in the degradation of pesticide residues. Most studies have shown promising outcomes, encouraging further research and scaling-up for commercialization. This review summarizes the use of cold plasma as an emerging technology for pesticide degradation in terms of the plasma system and configuration. It also outlines the key findings in this area. The most frequently adopted plasma systems for each application are identified, and the mechanisms underlying pesticide degradation using cold plasma technology are discussed. The possible factors influencing pesticide degradation efficiency, challenges in research, and future trends are also discussed. This review demonstrates that despite the nascent nature of the technology, the use of cold plasma shows considerable potential in regards to pesticide residue degradation, particularly in food applications.

## 1. Introduction

**Figure 1:**
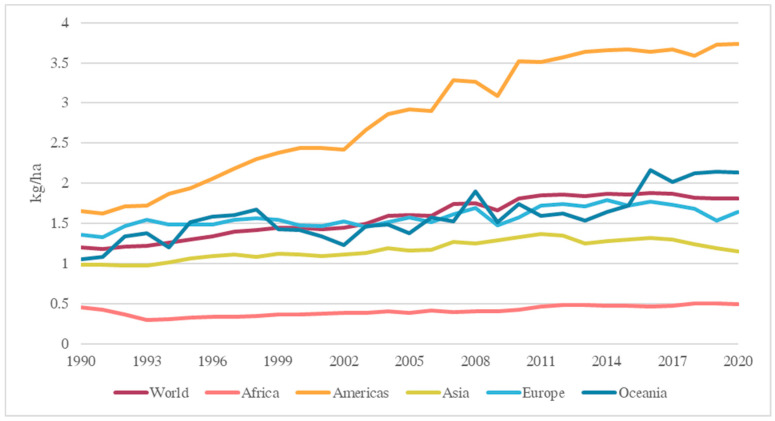
Regional pesticide usage per cropland area (Ritchie et al. [7]).

According to the United Nations, the world’s population is expected to reach 9.8 billion by 2050, and a record high 11.2 billion by 2100 [1]. With the increasing global population, the demand for land and other natural resources, including water, food, and agricultural products, will continue to rise [2]. To satisfy the rising demand for agricultural products, pesticides have been used to protect plants and crops from pests, insects, fungi, and weeds. In addition to agricultural applications, pesticides are used in human and animal hygiene products such as pet shampoos [3]; moreover, they are incorporated into building materials and boat bottoms to render them insect-resistant [4]. Pesticides, used before or after harvesting plants, contain both active and inert ingredients. They can be classified based on the type of pest targeted, such as herbicides, insecticides, rodenticides, and fungicides [5]. Pesticide usage has increased worldwide, except in Africa, since the 1990s. In 2020, America was the top contributor to the world’s pesticide usage, accounting for more than 50% of the world’s total (Figure 1). Pesticides have negative effects on water, soil, and animals [6].

Foods contaminated by harmful bacteria, viruses, parasites, or chemical substances, including pesticide residues, are consumed by an estimated 600 million people, which is approximately one-tenth of the world’s population, causing common foodborne illnesses and approximately 420,000 deaths annually [8]. Pesticides contained in food can accumulate in the human body, leading to chronic diseases. Additionally, polybrominated diphenyl ethers (PBDE), phthalates, bisphenol A (BPA), polychlorinated biphenyls (PCBs), and dioxins, as well as pesticides in the body, result in the generation of endocrine-disrupting chemicals (EDCs), which impair the normal function, biosynthesis, and biotransformation of hormones and the activity of endogenous hormone-metabolizing enzymes [9]. Exposure to pesticides can occur via contact with the skin, ingestion, or inhalation, and the severity of health issues can vary depending on the type of pesticide used, the duration and route of exposure, and individual health conditions, such as nutritional deficiencies and healthy/damaged skin [10]. The most frequently reported negative effects of pesticide exposure include Parkinson’s disease [11], gastrointestinal and respiratory issues [12], neurological issues [13], reproductive issues [14], and endocrine issues [15]. In addition to causing health issues, pesticides contaminate water and soil, causing environmental pollution.

Various techniques, such as washing [16,17,18], peeling [19], and thermal [20,21,22] and chemical [23] treatments, are used to remove pesticides in food before cooking. Washing with tap water is the easiest way to remove dust and other particles from food products [24]. Although this technique is simple, its efficiency depends on the pesticide concentration and type of washing operation employed [25]. Peeling is another method that can easily help remove pesticides because pesticides typically accumulate on the outer surfaces of fruits and vegetables [26]. However, this step will also remove some of the important vitamins or nutrients contained in the peels of some fruits and vegetables. Thermal treatments, including boiling, cooking, or sterilization, are used during food processing [25]. Although thermal treatment can effectively reduce the concentration of pesticide residues, some of the negative effects include nutritional loss when this method is applied to fruits and vegetables. Chemicals, such as chlorine dioxide, detergent solutions [27], and acetic acid [28], have been used to eliminate pesticide residues from food products. Similar to other conventional techniques, chemicals also affect the environment and human health. The aforementioned techniques are widely used in households owing to their simplicity; however, there are some concerns regarding their resulting environmental, sensory, and nutritional losses in practical applications. Therefore, various other promising technologies have been developed to improve the effectiveness of pesticide decontamination.

Emerging technologies based on pulsed electric fields (PEFs) [29,30,31], irradiation [32,33,34], high-pressure processing (HPP) [35], ultrasonication [36], ozonation [37,38], and cold plasma have recently been used in the food and agricultural fields for microbial inactivation and pesticide residues degradation. Although quality-related properties, such as nutritional properties, color, texture, and flavor of food, are unaffected by PEF, Gómez et al. [39] summarized the limitations of PEFs in meat and fish processing (e.g., high capital cost; inefficiency of spore inactivation; unavailability of commercial units in many regions of the world; the presence of bubbles, leading to nonuniform treatment, as well as operational problems; and the scarcity of economic and engineering studies regarding the feasibility of the upscaled continuous process). Similarly, the efficiency of pesticide removal by irradiation depends on the surface properties of the food products [25]. For instance, size, shape, and homogeneous surface characteristics should be considered to standardize the matrices of the samples [40]. The maintenance and system cost, as well as the lack of data on thermophysical properties under pressure, are the main limitations of the HPP technology [41]. Moreover, HPP could cause damage such as protein denaturation, color and structure changes, and metal ion release in certain foods [41,42,43]. Ultrasonic irradiation is an environmentally friendly technique that can help reduce pesticide residues [44]. The efficiency of this technique relies on the power, amplitude, and frequency of the ultrasonic waves [45]. Although ozonation is an efficient technique for reducing pesticide residues, consumer acceptability is poor owing to its toxicity [45]. Cold plasma is a nonthermal and advanced technology with no adverse effects on agricultural products after treatment. Plasma comprises several reactive species, free radicals, electrons, positive and negative ions, atoms, and molecules in the ground and excited states; the quanta of electromagnetic radiation show unique properties [46]. With these numerous reactive species, plasma technology has been employed in various applications such as micro-organism decontamination, microbial inactivation, sterilization, and pesticide degradation. In the past few years, many researchers have successfully applied plasma technology for degrading pesticide residues in foods [47], water [48], and soil [49]. Plasma-activated water (PAW) is generated by the interaction between cold plasma and water, and it has broad applications [50,51]. Many reviews have been published regarding the applications of PAW to various processes in agricultural activities, including microbial inactivation, seed germination, plant growth enhancement, and food preservation, as well as in the removal of pesticide residues [50,52]. The various reactive species generated in PAW play the main role in these applications. Without considering the type of working gas used, the following reactions occur during PAW treatment (Equations (1)–(4)) [53]:

(1) $$e+H_{2}O\to OH•+H+e,$$

(2) $$e+H_{2}O\to H_{2}O^{*}+e,$$

(3) $$e+H_{2}O^{*}\to H_{2}O^{+}+2e,$$

(4) $${H_{2}O+H}_{2}O^{+}\to OH•+H_{3}O^{+},$$

This review provides a framework of the use of cold plasma technology in the degradation of pesticides, including the possible factors influencing the degradation efficiency and related degradation mechanism and degradation products. Moreover, challenges and future trends are also discussed. This review aims to offer useful tools and information for understanding the potential of cold plasma technology in pesticide degradation applications, and the degradation mechanism is further studied. 

## 2. Types of Nonthermal Plasma

Nonthermal plasma (NTP) is a partially ionized gas comprising various types of atoms, molecules, and ions in excited and ground states, as well as reactive species and free radicals [54]. It can be produced through the electrical discharge of carrier gases under low or atmospheric pressure [55]. Any type of excitation energy can be used to ionize carrier gases, including air, oxygen, nitrogen, helium, hydrogen, argon, or even their mixtures, into a plasma state [46]. The common reactive species produced by nonthermal plasma are reactive oxygen species (ROS), reactive nitrogen species (RNS), and charged particles [56]. The concentration of these reactive species depends on the gases and liquids used, the chemical environment, the excitation voltage, and the mode of generation [57,58]. Plausible reactions regarding reactive species generation could be established using argon, oxygen, nitrogen, and air as the working gases (Table 1). These reactive species play an important role in various applications such as microbial inactivation, pesticide degradation, and seed germination. When applied to food products or media matrices, nonthermal plasma can be classified into two types: gas discharge and plasma-activated water (or any other solution).

### 2.1. Gas Plasma

**Figure 2:**
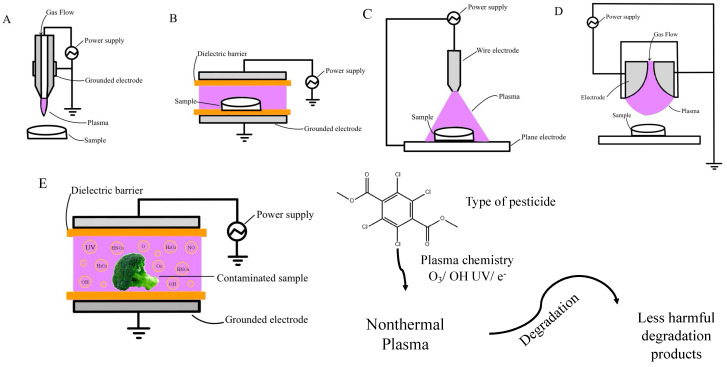
Plasma generation systems: atmospheric pressure plasma jet (APPJ) (**A**), dielectric barrier discharge (DBD) (**B**), corona discharge (**C**), gliding arc discharge (**D**), and pesticide degradation by nonthermal plasma (**E**).

As gas plasma or atmospheric-pressure cold plasma (APCP) can be produced at atmospheric pressure, it can overcome most of the drawbacks of conventional cold plasma, such as high investment cost, low processing speed, and the requirement of a vacuum system and special plasma reactor [62]. Plasma generation requires an appropriate plasma system that includes a carrier gas, a power source, and electrodes [46]. In this section, we discuss the various types of gas plasma discharges operating at atmospheric pressure in terms of the plasma generation method involved, including atmospheric-pressure plasma jet (APPJ) discharge, dielectric barrier discharge (DBD), corona discharge, and gliding arc discharge (GAD). Figure 2 provides an overview of the nonthermal plasma systems used for pesticide degradation.

#### 2.1.1. Atmospheric Pressure Plasma-Jet Discharge

Plasma-jet discharge is a promising technology for many applications, including material treatment [63,64,65], pesticide degradation [66,67], and microbial inactivation [68,69,70]. APPJ discharge is one of the most commonly used plasma systems owing to its versatility, low-cost tools, ease of design, and fewer requirements. However, it is unsuitable for large-area treatment because of its uniformity when applied to large areas [71]. This plasma system typically comprises a nozzle equipped with two electrodes in different arrangements, such as coaxial or special ring-electrode setups [62]. Plasma is produced through the nozzle and expands as a plume [72]. The voltage used to ignite the gas ranges from a hundred volts to kilovolts, depending on the gas type and discharge gap. The gases commonly used to produce plasma are air and gas mixtures of argon–oxygen and argon–nitrogen [73]. The gas flow rate is another design parameter influencing plasma generation in the APPJ discharge system. Table 2 summarizes the different applications of APPJ and its design parameters. Cold atmospheric plasma jets have been applied to improve the wettability of polypropylene (PP), in which pure argon is used as the working gas [74]. The contact angle, which indicates the wettability of the PP surface, decreases with the plasma treatment time. For surface modification via the plasma technology, the gas flow rate and the applied voltage have a considerable impact on the etching process [75]. Although various studies have indicated the impact of the plasma process parameters on different applications, the process parameters should be optimized for specific applications.

#### 2.1.2. Dielectric Barrier Discharge

The most frequently applied system for plasma technology is the DBD [81]. In this system, a dielectric material, such as plastic, quartz, or ceramic, is coated on the electrodes [82]. Air is also used in this system as a barrier to the current, preventing spark generation. The DBD system offers some key advantages, including simple a discharge ignition, the flexibility of using different gas mixtures, a lower gas flow rate requirement, and the possibility of using different electrode geometries and configurations to provide a uniform discharge ignition over several meters [83]. However, it requires a high voltage (10 kV) to ignite the plasma, which can be supplied by AC [84]. Design parameters, such as the type of round-edged electrodes, dielectric material, and applied voltage, should be appropriately selected to avoid arcing during plasma generation. The system uses typical carrier gases such as atmospheric air, nitrogen, argon, and helium [85]. Although the DBD system provides a large-area surface treatment, overcoming the constraints of APPJ discharge, limitations persist, such as the use of flat substrates and a small electrode gap. Moreover, the charge and average current density of the gas are limited owing to electrical breakdown [86]. Nevertheless, in the DBD system, the plasma discharge is randomly distributed, and a homogeneous treatment is ensured [87]. Table 3 lists the plasma applications through the use of DBD. Xiang et al. [88] inactivated *Zygosaccharomyces rouxii* in apple juice using DBD plasma; a 5-log reduction of viable *Z. rouxii* cells in apple juice with a DBD exposure time of 140 s was demonstrated. However, process optimization should be performed to realize an efficient and cost-effective inactivation process in the food industry. Samantaet et al. [89] investigated the effect of DBD plasma on the hydrophobic functionalization of cellulosic fabric, in which the plasma discharge voltage, the gas ratio of the gas mixture, and the gas flow rate significantly affected the fragmentation. This study demonstrated the complexity of plasma applications, which can be controlled by selecting appropriate plasma parameters to achieve the desired functionality.

#### 2.1.3. Corona Discharge

When a high electric current is applied between two electrodes separated by a small gap, corona discharge, which generates small lightning bolts, is generated near the sharp-edge electrode [93]. The corona discharge system includes a high-voltage supply, electrodes, and sample-treatment chambers. The electrodes used in this system, such as a point, tip, thin wires, or plane with an imposed high voltage, are typically asymmetrical [55,94]. The electrodes can be made of copper, ceramic-coated stainless steel, or titanium [95]. Air, nitrogen, argon, a mixture of helium and oxygen, or argon and oxygen can be used as the working gas to generate the discharge [96]. Corona discharge possesses limitations, as it produces inhomogeneous discharge and has a small treatment area, requiring scale-up operations [97]. Furthermore, corona discharge can cause burn spots, discoloration, and oxidation when applied directly to the food surface because the active region is close to the point electrode or is limited to distances of millimeters [87]. Therefore, corona discharge is unsuitable for applications that require large-area surface treatments. Table 4 shows its usage in various applications, including the inactivation of virus and foodborne pathogens, nanocomposite coating, shelf-life extension, and the synthesis of carbon nanotubes.

#### 2.1.4. Gliding Arc Discharge

To reduce the temperature to the nonthermal level, GAD is generated by the deposition of a cold, atmospheric-pressure plasma onto surfaces using forced air [102]. DC and AC can be used as power sources in GAD to generate near-atmospheric pressure plasma. The plasma system comprises two or more metallic electrodes, along with an AC or DC high-voltage transformer. An electric arc is produced between the electrodes, generating a plasma plume when a high voltage is applied [103]. The gliding arc outperforms other types of plasma discharges given its lower current intensity, higher electron density, higher injection flow rate, and lower cost [63,104]. Considering the advantages of GAD systems, various applications of this system are summarized in Table 5. Kimet et al. [105] reported that water injection and air flow rates affect the concentration of the hydrogen peroxide produced, while the exposure time of GAD affects bacterial inactivation.

### 2.2. Plasma-Activated Water

PAW can be produced by discharging plasma into a specific amount of water or solution using various plasma sources, including plasma jets, DBD, corona discharge, and GAD [110]. DBD is the most widely used plasma source for PAW production [111,112,113,114]. ROS and RNS can be generated by exposing plasma to water, in which the plasma discharge is transferred from the plasma to the liquid at the gas–liquid interface, producing various primary and secondary species that play a crucial role in many applications [115]. Two types of RONS are produced in PAW: long-lived species (e.g., hydrogen peroxide (H_2_O_2_), nitrate (NO_3_^−^), nitrite (NO_2_^−^), ozone (O_3_)) and short-lived species (e.g., hydroxyl radicals (•OH), superoxide (O_2_^−^), singlet oxygen (^1^O_2_), nitric oxide (NO•), peroxynitrite acid (ONOO^−^), and peroxynitric acid (OONO_2_^−^)). The generation of these species depends on various parameters, including the gases and liquids used, the chemical environment, the excitation voltage, and the fabrication modes. PAW can be produced using two approaches that rely on the plasma–liquid interaction, i.e., plasma discharge over and under the water surface. The type and concentration of RONS in PAW vary depending on the plasma source, the generation approach, and the operational parameters. The differences between these two approaches lie in the chemical and physicochemical properties of the generated species [50]. 

#### 2.2.1. Discharge over Water Surface

When plasma is exposed over the water surface, the water composition of the plasma gas–water surface changes [116]. The major concern regarding plasma discharge over a water surface is the mass transfer from gas to water. Therefore, the reactor used to mix the gas and water should be optimized [117]. Some researchers have overcome this issue by increasing the contact area [118], enhancing the contact time, adding contact regions [119,120], and adjusting the plasma distance [121]. In this form of discharge, the energy efficiency is improved through the production of reactive species in the gas-phase plasma, which transfer to the liquid or form at the liquid–gas interface [122]. Table 6 presents the applications of PAW, in which plasma discharges over the water surface. The discharge duration for PAW production and the PAW treatment time influence the effectiveness of decontamination [123,124]. The longer reaction time between the reactive species and the microbes appears to be a possible reason for this enhancement. According to Kumaret et al. [125], different activated solutions show different effects on the cell viability of pancreatic cancer cells.

#### 2.2.2. Discharge under the Water Surface

In the case of plasma discharge under the water surface, the water and discharge responses have a combined effect, leading to a more powerful reaction and more reactive species [129]. As the oxygen content in water is higher than that in air, more oxygen-containing groups are produced, resulting in ROS and free electrons. According to Piskarev [130], the energy required to activate water is less than that required to produce plasma. Moreover, plasma discharge under water provides better results in terms of physicochemical properties, such as the conductivity and oxidation–reduction potential (ORP) [131]. A possible reason for the higher efficiency of plasma exposure under water than over the water surface is that a closed system can produce more reactive species in PAW [132]. Table 7 lists the application of plasma discharge under the water surface. The gas mixture is one of the parameters influencing the generation of the chemical compounds in PAW. Different gas mixtures or ratios cause different reactive radicals to be generated in PAW [133]. In addition to the dissolved substances in PAW, the pH level, electrical conductivity, and oxidative reduction potential are other parameters set for PAW generation [134,135]. Liuet et al. [136] found that direct plasma treatment with different feeding gases can produce different degrees of enhancement in the germination of mung beans.

Several reports revealed that the parameter settings used to generate both gas plasma and PAW are crucial in almost every application. Therefore, the relevant parameters for plasma generation should be considered and optimized. For instance, Kim, Lee, Puligundla, and Mok [70] investigated the effect of relative humidity on the generation of reactive species (i.e., CO, NO, and NO_2_ species). An increase in the relative humidity level was found to improve the efficiency of the corona discharge plasma jet for the inactivation of foodborne pathogens. Panda and Sahu [141] demonstrated that the plasma condition and rate of hydrogen production can be enhanced, depending on the electrode material used in the DBD plasma reactor. They also studied the plasma discharge characteristics by varying the discharge voltage and time, while maintaining a discharge gap of 1.5 mm.

## 3. Pesticide Degradation in Cold Plasma

Cold plasma technology, as discussed in the previous section, is an effective and innovative technology for various applications, including pathogen inactivation, seed germination, surface modification, and pesticide degradation. Over four million tons of pesticides have been used annually over the past decade [142]. Pesticide usage has significantly affected the quality of water, soil, and air, as well as crop production, causing toxicity, carcinogenicity, and mutagenicity in humans [143,144,145]. Kimet et al. [146] adopted APCP to degrade paraoxon and parathion on glass slides. In recent years, the applicability of cold plasma for pesticide degradation in water, soil, and food has been increasingly studied. 

### 3.1. Degradation of Pesticide Residues in Water

Water contaminated with pesticide residues in agricultural areas negatively affects the ecosystem [147]. Several studies have applied nonthermal plasma to remediate pesticides in wastewater treatment [148]. Cold plasma-assisted dissipation of pesticide residues in water has been reported since 2008 [149], and the degradation efficacy has been satisfactory. In most studies, pulsed corona discharge [150,151] and DBD [152,153,154] with a falling water film were adopted for a direct discharge inside, or in contact with, the liquid [148]. Table 8 shows the degradation of pesticide residues in water using cold plasma technology.

Cold plasma transforms the chemical structure of pesticides to a structure that is nontoxic or less toxic [155,156]. In a previous study, organic micropollutants (atrazine, chlorfenvinphos, 2,4-dibromophenol, and lindane) contaminating a solution were removed using two different nonthermal plasma reactors [157], both of which were based on DBD, with one operating as a planar reactor and the other operating as a coaxial reactor. The efficiency of pollutant removal from water depends on the initial concentrations of the organic matter and mineral salts. Pollutant dissipation increases with increasing plasma treatment time. Huet et al. [158] and Hu, et al. [159] studied other parameters, including the discharge power and air gap distance. Both studies concluded that hydroxyl radicals play the most important role in degrading both pesticides. The effects of various plasma-generated parameters, such as the input power, treatment time, and input voltage, were studied. Ref. [160] found that the degradation efficacy of nitenpyram pesticide improves with increasing input power. In addition, the mineralization of 2,4 dichlorophenoxyacetic acid from an aqueous solution could be enhanced by increasing the plasma treatment time [161]. Some studies have also investigated the effects of external factors, such as catalysts, conductivity, and pH, on improving the degradation efficacy. Appropriate amounts and types of catalysts can help improve the degradation process [156,160]. Reddy et al. [154] showed that nonthermal plasma combined with cerium oxide catalysts improved the mineralization of endosulfan from an aqueous medium. Similarly, Jović et al. [154] examined four catalytic systems (Mn^2+^/DBD, Co^2+^/DBD, Fe^2+^/DBD, and H_2_O_2_/DBD). A lower pH or an acidic condition were found to result in a greater amount of pesticide degradation in aqueous solutions [161]. Ozonation is a promising advanced oxidation process for pesticide dissipation; Bradu et al. [151] treated a solution with a combination of plasma and ozonation reactors and found that the combined treatment resulted in a more effective degradation than did ozonation alone. More recently, microplasma discharge water has been applied to remove organophosphorus and organochlorine pesticides [162]. The results revealed that different types of pesticides were degraded via different mechanisms and reactive species. Many pesticides have been investigated in aqueous solutions. The key parameters in these treatments were the mineralization efficiency and solution toxicity. However, different types of pesticides and plasma discharge systems require different configurations of the plasma settings, as well as their optimization.

**Table 8:** Summary of studies on the degradation of pesticide residues in water using cold plasma technology.

Pesticide	Plasma System	Plasma Configuration	Key Findings	Reference
2,4-dinitrophenol (DNP)	Dielectric barrier discharge	Working gas: air Input power: 150 W (AC source) Discharge time: 60 s Voltage: 100 V Dielectric barrier: quartz	Degradation value: 83.6% Fe^2+^ is conducive to DNP degradation The pH value decreases with increasing discharge time.	[149]
Atrazine, chlorfenvinphos, 2,4-dibromophenol, and lindane	DBD (a conventional batch reactor)	Dielectric barriers: Pyrex glass containers Working gas: helium Frequency: 100 kHz Power: 30 W Voltage: 20 kV Distance between both electrodes: 16 mm High-voltage electrodes: metallic cylinders	Kinetic constant (*k*) 0.534 min^−1^ for atrazine 0.567 min^−1^ for chlorfenvinphos 0.802 min^−1^ for 2,4-dibromophenol 0.389 min^−1^ for lindane The efficiency declines when the solution to be treated contains high concentrations of organic matter and mineral salts.	[157]
	DBD (a coaxial thin-falling water-film reactor)	High-voltage electrode: copper mesh Dielectric barrier: glass vessel Grounded electrode: stainless-steel tube Working gas: helium High-voltage DC pulses: 12 kV Power: 24 W Repetition frequency: 94 kHz	Kinetic constant (*k*) 0.104 min^−1^ for atrazine 0.523 min^−1^ for chlorfenvinphos 0.273 min^−1^ for 2,4-dibromophenol 0.294 min^−1^ for lindane	
Dimethoate	Dielectric barrier discharge	Applied power: 85 W Airgap distance: 5 mm Current: 0–1.2 A Voltage: 0–250 V Frequency: 5–35 kHz Electrodes: stainless steel Dielectric barrier: quartz plate	Degradation efficiency: >96% The degradation efficiency is improved by adding radical promoters. The hydroxyl radical (•OH) plays an important role in the degradation pathways.	[159]
Dichlorvos and dimethoate	Dielectric barrier discharge	Frequency: 5–35 kHz Voltage: 0–250 V Current: 0–1.2 A Electrodes: stainless steel Dielectric barrier: quartz plate	The degradation efficiency increases with a higher discharge power and a shorter airgap distance. Hydroxyl radicals are most likely the main drivers of the degradation process.	[158]
Nitenpyram	Dielectric barrier discharge	Optimum voltage: 80 V Current: 1–2.5 A Dielectric barrier: quartz glass Distance between the barrier and the solution surface: 8 mm Input power: 200 W Treatment time: 180 min	NTP can be effectively removed from the aqueous solution. Increasing the input power improves the degradation efficiency. A suitable catalyst improves the degradation process. The pH of NTP reduces with discharge time. Decomposition of NTP: 82.7%	[160]
Mesotrione	Dielectric barrier discharge	Dielectric barrier: glass tube Inner electrode: stainless steel Outer electrode: stainless-steel mesh Apply voltage: 17 kV Frequency: 300 Hz Power: 65 W	Catalytic systems are more efficient than noncatalytic DBD treatment. Most efficient catalytic system: 5 ppm Fe^2+^/DBD Highest mineralization efficiency (71%): system 10 mM H_2_O_2_/DBD In terms of global toxicity, samples after degradation in each catalytic system can be considered nontoxic.	[156]
Endosulfan	Dielectric barrier discharge	The gap between electrodes: 3.5 mm Inner electrode: stainless-steel rod Ground electrode: silver plate Dielectric barrier: quartz tube Voltage: 1–40 kV Working gas: air	Best performance: adding catalyst CeO_2_ The conversion increases with a higher input power, but decreases with increasing ES concentration. Conversion rate: 82% Mineralization: 15% The combination of cerium oxide catalyst increases the conversion to 94% and the mineralization to 48%.	[154]
Dichlorvos, malathion, and endosulfan	Dielectric barrier discharge	Working gas: atmospheric air Electrodes: aluminum plate Input voltage: 230 V Frequency: 50 Hz Dielectric barrier: polypropylene container	Degradation efficacy 78.98 ± 0.81% for dichlorvos 69.62 ± 0.14% for malathion 57.71 ± 0.58% for endosulfan The degraded compounds and intermediates formed were less toxic than the parent pesticide.	[155]
Chlorophenoxyacetic herbicide 2,4-D	Pulsed corona discharge	Working gas: oxygen Solution layer depth: 5 mm Pulse repetition rate: 25 Hz High-voltage electrode: copper wire	Apparent reaction rate: 0.195 min^−1^ Mineralization: more than 90% after 60 min Performance enhancement is attributed to the formation of other reactive oxidizing species besides the ozone. Improvement in the energy efficiency: optimization of the electrical characteristics of the discharge.	[151]
Bisphenol A (BPA), estrone (E1), and 17b-estradiol (E2)	Dielectric barrier discharge	Working gas: air Electrode: aluminum plate High-voltage electrode: acrylic sheet Input voltage: 230 V Frequency: 50 Hz	Degradation efficiency 93% for BPA 83% for E1 86% for E2 Oxygen radicals play a key role in the degradation process.	[153]
2,4-dichlorophenoxyacetic acid	Pulsed corona discharge	Water depth: 2 cm Height of high-voltage electrodes: 5 mm above liquid Pulse voltage: 140 kV	A higher degradation of 2,4-D was observed under acidic pH conditions. Toxicity: 10 mg/L Complete degradation was within 6 min with a yield of 0.9 g/kWh	[161]
Carbamate (carbaryl, methiocarb and aminocarb)	Dielectric barrier discharge	Optimal voltage: 90 kV Optimal duration: 5 min Working gas: dry air Electrodes: circular aluminum plate Dielectric barrier: Plexiglass and polypropylene Distance between the electrodes: 49 mm	Maximum degradation 50.5% in carbaryl 99.6% in methiocarb 99.3% in aminocarb	[152]
Organophosphorus pesticides (chlorpyrifos, chlorpyrifos oxone, and diazinon) and an organochlorine pesticide (DDT solution)	Microplasma discharge water	Applied voltage: 30 kV Power ingestion: 153.7 ± 0.57 W Working gas: air	Nitrogen oxide plays the main role in degrading organophosphorus pesticides. Dissolved ozone and hydroxyl radical play a key role in the degradation of organochlorine pesticide. Degraded pesticide molecules transform to several smaller molecular components	[162]
Dimethoate	Plasma needle	Working gas: argon Power supply: 2.5 kV The tip of the power electrode: 5 mm below the surface of sample Gas flow rate: 0.5 slm Treatment time: 30 min	Dimethoate reduction: 1 × 10^−4^ M Degradation product: dimethoate oxo-analogue omethoate The degradation product is more toxic than parent dimethoate.	[163]

### 3.2. Degradation of Pesticide Residues in Soil

Soil is known to be an important resource in the agricultural field [164]. One of the major threats to soil is the diffusion of pesticides into soil layers, which hinders several of the United Nations Sustainable Development Goals related to the soil environment [165]. Pesticide residues in soil can be removed by washing [166] and volatilization [167]. However, this causes water and air contamination. Various effective methods, including bioremediation, chemical remediation, and electrokinetic remediation, have been explored to treat soil contaminated with pesticides or other compounds [168]. Although these techniques can remediate contaminated soil, some drawbacks remain, such as slow treatment and higher treatment costs. Plant-microbial remediation is one of the most effective methods for dissipating pesticide residues in soil. However, this method is limited when it comes to actual applications due to its high cost [169]. Hence, cost-effective cold plasma technology has been examined to remove pesticide residues in soil. p-Nitrophenol (PNP) was removed from contaminated soil using pulsed-discharge plasma combined with TiO_2_ [170]. Higher quantities of active species, such as O_3_ and H_2_O_2_, were observed in the pulse discharge plasma–TiO_2_ catalytic system than in a system using plasma only. The improvement was evident from the evolution of the main intermediates over the treatment time. Table 9 summarizes previous studies on the effects of cold plasma on the degradation of pesticide residues in soil. Pulsed corona discharge and DBD have been extensively applied to dissipate pesticide residues in soil. Planar or cylinder-to-plane electrode configurations of the DBD plasma discharge system are typically used, whereas for corona discharge, a multi-needle-to-plate configuration is preferred for soil remediation [171]. Wang et al. [166] investigated the effects of several parameter settings of the pulsed corona discharge plasma, including the peak pulse voltage, pulse frequency, gas atmosphere (air, O_2_, Ar, and N_2_), air flow rate, and pollution time, on the effectiveness of pentachlorophenol (PCP) degradation in soil. These results indicate that the degradation efficiency can be enhanced by increasing the peak pulse voltage or pulse frequency. Similarly, the discharge voltage of atmospheric-pressure DBD plasma also has a positive effect on the degradation efficiency of glyphosate-contaminated soil [172]. Although the plasma discharge systems were different, the plasma parameters, such as the voltage, still play an important role and should be optimized before application. Moreover, soil remediation decreased with an increase in the initial pollutant concentration. In the removal of residues from the soil, the ozone is the main active species in the gas form. Other parameters, such as the treatment time and the soil pH, also have a positive effect on pesticide extermination [173]. Soil moisture has been found to influence the remediation efficiency of plasma technology [49,172]. The latest report by Ref. [174] confirmed the effect of soil moisture on the pesticide degradation efficiency; however, no significant correlation was found with the soil thickness. According to previous studies, the properties of soil, particularly its moisture content, influence the soil remediation process, in which the reaction between the reactive species generated by plasma and water molecules produces strong oxidizing agents including ·OH and H_2_O_2_ [171]. 

### 3.3. Degradation of Pesticide Residues in Food

Food safety, nutrition, and food security are indistinguishably connected. Recently, these have become topics of major concern worldwide. One of the keys to sustaining life and promoting good health is accessibility to sufficient safe and healthy food. Unsafe food containing harmful bacteria, viruses, parasites, or chemical substances results in more than 200 diseases, ranging from diarrhea to cancer [178]. Conventional methods, such as heat treatment, chlorine treatment, ultrasound application, and ozonation, have been used to remove pesticide residues from food products. However, these techniques have various negative effects on the physical and chemical qualities of food products, along with environmental impacts. Cold plasma technology is a proven degradation process for pesticide residues found in food products owing to its minimal effects on product quality and attributes. Table 10 presents the applicability of cold plasma for removing pesticide residues from food products. Various types of food, such as blueberries, strawberries, cucumbers, and maize, were examined. Using the O_2_ plasma treatment, Bai et al. [179] demonstrated that the chemical structure of pesticides transforms into a structure with less toxic compounds, and this was confirmed by gas chromatography–mass spectrometry (GC/MS) analyses. A nontoxic secondary metabolite of chlorpyrifos, 3, 5, 6-trichloropyridinol, was observed after plasma treatment [180]. This chlorpyrifos degradation product was less harmful than the parent chlorpyrifos [181]. Furthermore, an in-package nonthermal plasma technology was first examined by Misra et al. [182]. Strawberries were exposed to a range of pesticides and then treated with the DBD system. The results showed a range of degradation percentages between 45% and 71%, depending on the type of pesticide used. Different types of pesticides have different chemical structures; therefore, the degradation efficiency of the plasma technology differs. The operating parameters of the atmospheric-pressure air DBD plasma system, including the plasma treatment time, discharge power, and initial pesticide concentrations, are other factors affecting the degradation efficiency [183]. Cong et al. [184] investigated the efficiency of dielectric barrier discharge on the degradation of malathion and chlorpyrifos in water and on lettuce at different voltages (60, 70, and 80 kV) and different times (30, 60, 90, 120, 150, and 180 s). An optimum treatment condition for pesticide degradation in water was a voltage of 80 kV and a treatment duration of 180 s. Interestingly, ascorbic acid significantly decreased when the long duration required for optimum treatment was applied. Therefore, a shortened treatment duration of 120 s was suggested to maintain the amount of ascorbic acid in lettuce. Several studies have determined the impact of cold plasma on the quality of fresh produce, such as its color, hardness, and sugar percentage. Most studies have shown that cold plasma has minimal undesired effects on food products, and that it can improve some of its chemical qualities [185,186,187]. Plasma-activated water, a novel treatment strategy, was applied to reduce phoxim on grapes [139]. After a 10 min treatment with PAW prepared for 30 min, phoxim was reduced by 73.60%. The experimental results also revealed that PAW treatment did not significantly affect the quality of the grapes, including color, firmness, sugar, vitamin C, and superoxide dismutase. In contrast, a decrease in the firmness of cherry tomatoes and blueberries was observed. This was the effect of direct treatment with nonthermal plasma on agricultural products, which is attributed to surface softening and mechanical damage, damage to external cells, and an increase in temperature during treatment [188,189]. Liu et al. [190] also found that plasma treatment for 60 s at an air flow rate of 1000 mL/min, power of 20 W, and frequency of 1200 Hz can remarkably decrease (*p* < 0.05) the moisture content of treated corn, while dissipating chlorpyrifos and carbaryl by up to 86.2% and 66.6%, respectively. Some studies have shown that the surface properties of fruits are affected by plasma penetration [191]. The analyses of the efficiency of pesticide degradation in grapes and strawberries using PAW showed chlorpyrifos degradation rates of 79% and 69% for grapes and strawberries, respectively, whereas the carbaryl degradation rates were 86% and 73%. Sawangrat et al. [192] adopted a pinhole plasma jet to generate PAW; the carbendazim and chlorpyrifos contained in the solution and on the chili surface was subsequently degraded. For the same plasma configuration, they reported that the pesticide degradation efficiency was higher on the chili surface than in the pesticide solution. However, when fresh produce was treated with PAW, it required an additional drying process after treatment to avoid the wet produce decaying in storage. Overall, the efficiency of pesticide degradation from food products using cold plasma depends on several factors, including plasma configuration, initial concentration of pesticide residue, surface characteristics of food products, and type of pesticide, as well as environmental factors.

## 4. Mechanism of Nonthermal Plasma on Pesticide Degradation

For a better understanding of the reactions by which the plasma treatment degrades pesticides, plasma chemistry, such as the produced reactive species and degradation products, should be investigated. Some studies have found that the reactive species components [155] and electron energy [46] are important factors indicating the potential of pesticide degradation. Plasma treatment could produce various high-oxidation-potential reactive species, such as ozone, hydroxyl radical, and hydrogen peroxide, as well as irradiated light and ultraviolet light [48]. The presence of these reactive species appears to play a critical role in the degradation mechanism [179]. Direct oxidation or several chain reactions that yield H_2_O_2_ and the OH radical are the main mechanisms of ROS for pesticide degradation [193]. At a high pH, an indirect reaction occurs, whereas a direct reaction is predominant in acidic environments. In acidic environments, a slow reaction between the dissolved ozone and hydrogen peroxide occurs, leading to the formation of the hydroxyl radical. However, these reactions are significantly accelerated at high pH levels. Ozone also leads to oxidation by cleaving double bonds and direct reactions with compounds such as OH, CH_3_, OCH_3_, and NH_2_. A few chemical reactions that may occur during and post-plasma treatment are presented in Equations (5)–(12) [155].

H_2_O + e^−^ → H^•^ + OH^•^ + e^−^, (5)

O + O_2_ → O_3_, (6)

H_2_O + e^−^ → H• + OH• + e^−^, (7)

O_3_ + ^•^OH → ^•^HO_2_ + O_2_, (8)

OH^•^ + OH^•^ → H_2_O_2_, (9)

H^•^ + O_2_ → HO_2_, (10)

HO^•^_2_ + H^•^ → H_2_O_2_, (11)

H_2_O_2_ + O_3_ → ^•^OH + O_2_ + ^•^HO_2_, (12)

**Figure 3:**
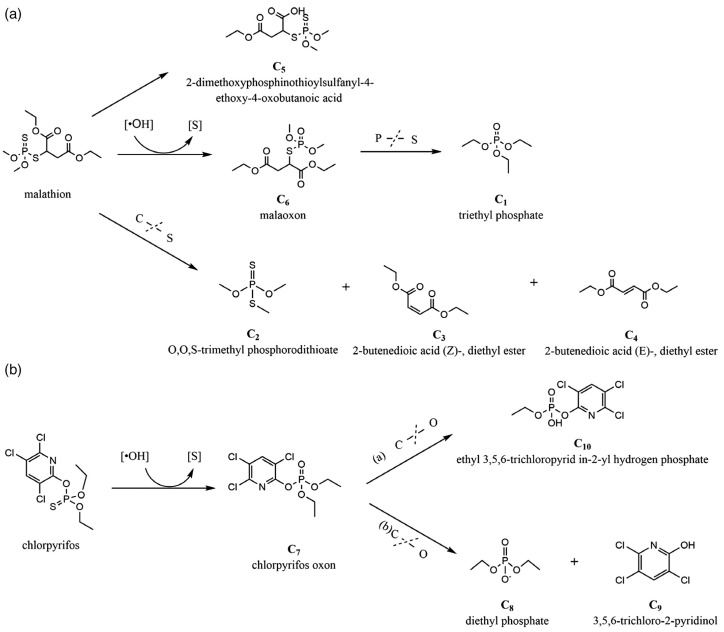
Degradation pathways of (**a**) malathion and (**b**) chlorpyrifos by the DBD plasma (Copyright 2020, Society of Chemical Industry [184]).

Based on the effect of plasma treatment on pesticide residues, RONS directly attack and react with the pesticide molecules, breaking chemical bonds and forming various chemical reactions that lead to the transformation of pesticide structures into less harmful or harmless compounds under suitable conditions [199]. The P=S double bonds in dimethoate are first cleaved via a hydroxyl radical attack. Finally, they are transformed into small harmless compounds containing groups including PO_4_^3−^, H_2_O, and CO_2_ [159]. In the application of pulsed corona discharge above the water surface for the degradation of carbofuran and 2,4-dichlorophenoxyacetic acid (2,4-D) [200], the oxidation mechanism initiated by the hydroxyl radical leads to the transformation of carbofuran and 2,4-D to carbamic acid and 2–4-dichlorphenol, respectively. Another crucial property of the degradation pathway is the hydroxylation of the C–O bonds on the benzene ring by the hydroxyl radicals. Ozone, which is a powerful oxidant, can be produced at industrial scales by electrical discharge in oxygen and in air by corona or the DBD discharge system [199]. Chamberlain et al. [201] found that the degradation mechanism of pesticides can be attributed to the photolysis reaction by the hydroxyl radicals and oxidation–reduction reactions by ozone and oxygen atoms. Small molecular compounds, including acids, alcohols, amines, and carbonyls, are the by-products of oxidation from the ozone [202]. Khan et al. [203] explained the mechanism of diazinon degradation in microplasma discharge water. The various radicals generated in PAW, particularly the oxide of nitrogen and ozone, played various roles in the degradation mechanism. Smaller molecular fragments, including hydroxy diazinon and isopropenyl derivative, were formed through hydroxylation and dehydration reactions, respectively. Unlike other research, this study showed that hydrogen peroxide has a minimal effect on the degradation pathways of diazinon [203]. Bennett et al. [204] compared the efficiencies of two plasma systems based on GAD and pinhole plasma discharge. Over 50% of diruron was degraded with pinhole plasma discharge, whereas the GAD was insufficient for diuron degradation. The degradation products of diuron were identified to be 3,4-dichloro-benzenamine, 1-chloro-3-isocyanato-benzene, and 1-chloro-4-isocyanato-benzene through GC-MS; some authors suggested that these degradation products are more harmful to the environment than the parent compound [205]. Similarly, Mitrović et al. [155] investigated the effect of a nonthermal plasma needle on the removal of dimethoate from water. According to the high-performance liquid chromatography (HPLC) analysis, one of the degradation products, dimethoate oxo-analogue omethoate, is more harmful than dimethoate. However, the overall toxicity of contaminated water continuously decreases after treatment. Cong et al. [182] proposed a degradation pathway of malathion and chlorpyrifos by DBD plasma (Figure 3). A P=S double bond is first destroyed, and then it is transformed to the P=O double bond in malathion and chlorpyrifos. Subsequently, melaoxon is converted to triethyl phosphate by the cleaving of the P-S bond. Simultaneously, the molecules of malathion can degrade into O,O,S-trimethyl phosphorodithioate, 2-butenedioic acid (Z)-, diethyl ester, 2-butenedioic acid (E)-, and diethyl ester by breaking the C–S bond attacked by active species. For the chlorpyrifos degradation, the oxidation product of chlorpyrifos oxon is further converted into diethyl phosphate, 3,5,6-trichloro-2-pyridinom, and ethyl 3,5,6-trichloropyrid in-2-yl hydrogen phosphate by the cleaving of the C–S bond. From these results, we conclude that the degradation mechanism differs, depending on the pesticides added to a specific food product or media matrix and the specific plasma discharge system used, and should therefore be investigated specifically for each pesticide and system. 

## 5. Conclusions and Future Directions 

**Figure 4:**
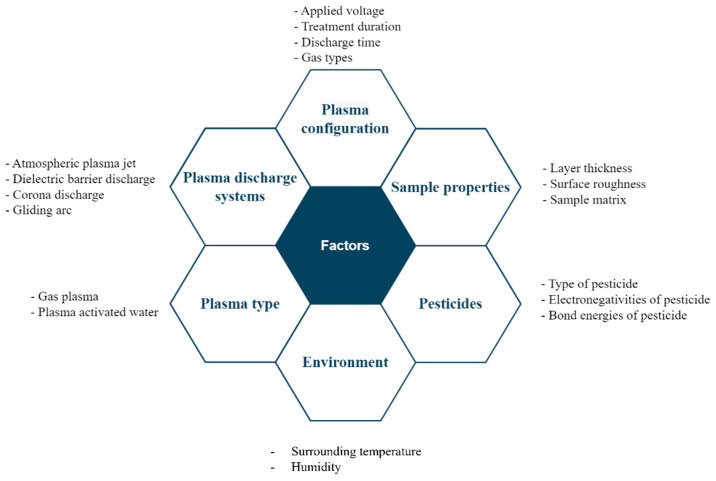
Effect of pesticide degradation factors using cold plasma treatment.

Pesticide usage is increasing, resulting in chemical residues in the environment. Although several existing techniques can be used to address this issue, they all exhibit potential, along with adverse effects. Owing to its promising efficiency in several applications, including pesticide degradation, the cold plasma technology is currently being extensively studied. Various plasma-generation techniques, including APPJ, DBD, corona discharge, and GAD, have been proposed. These systems can generate plasma in gas or liquid forms. However, not every system is applicable in every scenario. The APPJ is suitable for flat and small samples, whereas the DBD method can be applied to samples with a large surface area. Similar to APPJ, corona discharge, which produces an inhomogeneous discharge, possesses a small treatment area. Recently, GAD has been widely studied in various applications owing to its low current intensity, low cost, high electron density, and high injection flow rate. However, few studies have adopted GAD for pesticide degradation. Therefore, there remain various gaps that should be addressed by the research community. For example, an appropriate plasma generation technique and its optimal setting condition should be verified to obtain the maximum degradation rate regarding specific pesticides and samples. For implementation in a wide range of industries, optimizing cold plasma treatments for specific degradation applications (i.e., pesticides, food products, or solutions) is challenging and should be explored by stakeholders, including researchers. Figure 4 illustrates the factors influencing the pesticide degradation efficiency. This point should be addressed to obtain the most desirable results when plasma treatment is applied to pesticide degradation. Several plasma configuration parameters, such as the discharge time, feeding gas types, treatment time, power, and applied voltage, affect the degradation potential. Factors including the sample properties, pesticide types, and the environment also affect the pesticide degradation efficiency. Therefore, more experimental designs should be adopted to define suitable plasma parameters. Although plasma treatment has been considered a promising technique for pesticide degradation, it does not fully remove pesticides. Hence, its incorporation with other technologies, including microbubbles, UV light, ozone, or shockwaves, may be an alternative to improve the degradation efficiency.

Research on cold plasma technology for pesticide degradation in fresh produce is still limited in terms of the penetration depth of plasma-generated reactive species, the effects of cold plasma on quality attributes, and its mechanism, as well as the toxicity of the degradation products. Although some studies have proposed possible pesticide degradation pathways and degraded products, the specific reactive species and their interaction on the pesticide structure are yet to be fully explored. Most studies have shown that hydroxyl radicals and ozone play an important role in the degradation of pesticides. Food safety is another concern associated with this technology; hence, the final by-products and possible side effects of plasma treatment should be considered to gain the trust of consumers. Despite these limitations, this review proves that cold plasma technology exhibits high potential for removing pesticide residues in various samples, particularly in food applications. The potential to upscale and expand cold plasma technology in the food industry should be considered in further investigations.

## Figures and Tables

**Table 1:** Plausible reactions obtained from the interaction between plasma and water molecules when using different working gases.

Working Gas	Reaction	References
Argon	$Ar+e\to {Ar}^{+}+e+e$	[59]
	$Ar+e\to {Ar}^{*}+e$	
	${Ar}^{*}+H_{2}O\to Ar+OH•+H$	
Oxygen	$O_{2}+e\to O^{+}+O+2e$	[60]
	$O_{2}+e\to O^{-}+O$	
	$O+O_{2}\to O_{3}$	
	$O+H_{2}O\to 2OH•$	
	$O_{3}+OH^{-}\to HO_{2}^{-}+O_{2}$	
	$O_{3}+HO_{2}^{-}\to OH•+O_{2}^{-}+O_{2}$	
Nitrogen	$e+N_{2}\to N+N+e$	[60]
	$e+N_{2}\to N_{2}^{*}+e$	
	$N^{*}+H_{2}O\to N+OH•+H$	
	$N^{*}+H_{2}O\to OH•+NH$	
	$N_{2}^{*}+H_{2}O\to N_{2}+H+OH•$	
	$H_{2}O+h\upsilon \to OH•+H$	
Air	$e+N_{2}+O_{2}\to 2N+2O+e$	[61]
	N+O→NO•	
	$N+O_{2}\to NO•+O$	
	$O+N_{2}\to NO•+N$	
	$H_{2}O+h\upsilon \to OH•+H$	

**Table 2:** Applications of cold plasma produced by an atmospheric-pressure plasma jet (APPJ).

Gas Type	Design Parameter	Applications	References
Ar	Voltage: 6 kV Frequency: 60 Hz Argon gas flow: 3 slm Exposure time: 1.7 min	Spore inactivation	[76]
Ar	High-voltage power supply: 5.5 kV Frequency: 20 kHz Gas flow rate: 3 L/min Temperature: 27 °C	Wettability improvement	[74]
Air	Power: 400–800 W Exposure time: 30 min	Corn starch modifications	[77]
Ar	Voltage: 16 kV Frequency: 24 kHz Argon flow rate: 2 slm Distance between the tube and treated seed: 2 mm Exposure time: 10 min	Seed germination	[78]
He + O_2_	Applied voltage: 17–20 kV_pp_ Frequency: 5 kHz Helium gas: 1–2 standard liters per minute (slm) Oxygen gas flow rate: 0.01–0.08 slm Distance of jet from sample: 10–30 mm	Antibacterial effects	[79]
Ar + N_2_	Deposition time: 30 or 60 s Distance between the nozzle and substrate: 53 mm Power: 360 W Frequency: 20 kHz	Surface insulation performance improvement	[80]
He + O_2_	Helium flow rate: 200 sccm Oxygen flow rate: 0–30 sccm Voltage: 8–18 kV Frequency: 20 kHz Distance between the tube and film surface: 1 mm Sample etching time: 40 s	Polymer film treatment	[75]

**Table 3:** Plasma application through DBD.

Gas Type	Configuration	Applications	References
Air	Input power: 90 W Dielectric barrier: quartz plate Electrodes: steel Treatment time: 140 s Gap distance: 10 mm	Inactivation of yeast spoilage	[88]
N_2_ + air	Power: 400 W Plasma-generating area: 91.875 cm^2^ Electrode area: 110.25 cm^2^ Dielectric barrier: lumina ceramic plate	Seed germination and plant growth	[90]
Helium/tetrafluoroethane	Voltage: 6.0 kV Frequency: 17.4 ± 0.74 kHz Dielectric material: Teflon or glass Gap distance: 2.1 mm Electrode material: aluminum	Hydrophobic functionalization of cellulosic fabric	[89]
Air	Dielectric barrier: alumina ceramic plates Gap distance: 1 mm Power source: 10 kV, 12 kHz	Soil treatment	[91]
Argon	Distance between electrodes: 10 mm. Peak-to-peak voltage: 0–11.86 kV Dielectric barrier: circular acrylic plate Electrode: stainless steel	Bacterial reduction	[92]

**Table 4:** Plasma application through corona discharge.

Carrier Gas Type	Configuration	Applications	References
Gas mixture (CH_4_ + H_2_)	Upper electrode: tungsten wire Lower tungsten: circular-plate stainless steel Voltage: 8 kV Frequency: 25 kHz Power 40 W	Synthesis of carbon nanotubes	[98]
Air	Output voltage: 8 kV Frequency: 20 kHz Electrode: ring-shaped stainless steel	Improving microbial quality and shelf life	[99]
Air	High voltage: 20 kV Input current: 1.5 A Frequency: 58 kHz High-voltage electrode: tungsten	Inactivation of foodborne pathogens	[70]
Helium	Frequency: 27 kHz High-voltage electrode: tungsten Voltage: 1.8–2.2 kV Discharge power: 40–90 W Gas flow rate: 0–1 L/min	Deposition of nanocomposite coatings	[100]
Air	Low-voltage electrode: stainless-steel circular plate High-voltage electrode: stainless-steel multi-needle Peak voltage: 19 kV Frequency: 80 Hz	Virus inactivation	[101]

**Table 5:** Plasma application through gliding arc discharge.

Gas Type	Configuration	Applications	References
Air	Power supply: 200 W Electrode: circular stainless-steel disk Gap between electrodes: 2.5 mm Maximum voltage: 3 kV	Inactivation of *Escherichia coli*	[105]
Compressed air	Frequency: 50 Hz Power input: 750 W Electrode: copper	Hydrophobization of cotton fabric	[106]
Argon	Voltage: 8 kV Power: 600 W Current: 0.6 A	Retardation of mango anthracnose	[107]
Compressed dry air	Voltage: 10 kV Frequency: 50 Hz Electrode: stainless steel Power: 500 W	Modification of polypropylene	[108]
Air	Electrode: stainless steel Gap between electrodes: 8.16–20.18 mm Gas flow rate: 10 L/min Frequency: 50 Hz Applied power: 300 W Peak-to-peak voltage: 27 kV	Drying efficiency	[109]

**Table 6:** PAW applications (discharge over water surface).

Gas Type	Configuration	Applications	References
Argon/Oxygen (Ar:O_2_ = 98%:2%)	Plasma system: plasma jet High-voltage source: 18 kV peak-to-peak voltage Frequency: 10 kHz Flow rate: 5 L/min Distance from the liquid surface: 2 cm	Inactivation of foodborne pathogens on strawberries	[122]
Argon gas	Plasma source: plasma jet High-frequency sinusoidal voltage: 2–6 kVp-p Frequency: 2.5 MHz Maximum power: 3.5 W Gas flow rate: 3 l pm Distance between the nozzle and water surface: 10 mm	Inactivation of human pancreatic ductal adenocarcinoma	[125]
Air	Plasma system: corona discharge Pin-electrode: stainless steel Distance from the liquid surface: 5 mm Peak voltage: 9 kV Frequency: 5 kHz	Decontamination and nutritional value	[126]
Compressed air	Plasma system: plasma jet Input power: 295 V Frequency: 22.5 kHz Distance from the liquid surface: 5 cm	Inactivation of *E. coli* and *Listeria innocua*	[123]
Room air	Plasma system: surface barrier discharge Gap between the liquid and electrode: 44.8 mm Frequency: 18 kHz	Food packaging	[127]
Air	Plasma system: DBD Dielectric barrier: aluminum oxide (Al_2_O_3_) Power: 51.7 W Frequency: 14.4 kHz Voltage: 8 kV	Inactivation of aerobic bacteria and coliform bacteria	[124]
Atmospheric air	Plasma system: spark discharge Resonance frequency: 60 kHz Duty cycle: 50 μs Electrode: copper	Nutritional composition, storage quality, and microbial safety	[128]

**Table 7:** Applications of PAW (discharge under the water surface).

Gas Type	Configuration	Applications	References
Air	Plasma system: plasma jet Current: 1.1–1.3 mA Voltage: 8.2 kV Air flow rate: 1.2 L/min Inoculation time: 30 min Activation time: 60 min	Inactivation of yeast on a grape	[137]
Air	Plasma source: DBD Flow rate: 1.0 L/min Peak voltage (Vp): 0–20 kV AC frequency: 9 kHz	Maintaining the antioxidant activity	[138]
Air	Plasma system: plasma jet Peak voltage: 25 kV Frequency: 20 kHz	Pesticide residue reduction	[139]
N_2_, O_2_, and air	Plasma system: DBD High-voltage electrode: stainless-steel wires Gas flow rate: 1.5 slm Ground electrode: annular aluminum	Enhancement of seed germination	[136]
Ambient air and compressed N_2_	Plasma source: plasma jet Gas flow rate: 1 L/min Discharge time: 10 min Temperature: 150–200 ℃	Beef curing	[140]
Air	Plasma system: APPJ discharge Voltage: 3.0 kV Frequency: 16 kHz Power: 60 W	Antibacterial activity	[134]
Atmospheric-pressure air	Plasma system: DBD High-voltage electrode: copper spring Grounding electrode: copper mesh Discharge voltage: 2.8 kV Frequency: 10 kHz	Microbial inactivation	[135]
N_2_ + O_2_	Plasma system: piezoelectric direct discharge plasma Power: 60–70 W Air flow rate: 20 L/min Activation time: 20 min	Glazing agent on a shrimp	[133]

**Table 9:** Summary of studies on the degradation of pesticide residues in soil using cold plasma technology.

Pesticide	Plasma System	Plasma Configuration	Key Findings	Reference
Pentachlorophenol (PCP)	Pulsed corona discharge	Working gas with optimal efficacy: oxygen High-voltage pulses: 0–50 kV Pulse frequency: 0–150 Hz High-voltage electrode: nine stainless-steel hypodermic pinheads Ground electrode: wire netting Distance between electrodes: 12 mm	The degradation increases with an increase in the peak pulse voltage or pulse frequency. The ozone plays an important role in PCP degradation. Maximum PCP degradation efficiency: 92%	[175]
*p*-Nitrophenol (PNP)	Pulsed discharge plasma	Catalyst: TiO_2_ Optimum amount of TiO_2_: 2% Pulse frequency: 100 Hz Pulsed discharge voltage: 20 kV Pulse-forming capacitance: 200 pF Input energy per pulse: 0.023 J	PNP degradation: 88.8% Higher TiO_2_ amount has an inhibitive effect. A higher air moisture content enhances PNP removal.	[176]
Contaminant mixture containing *p*-nitrophenol and pentachlorophenol	Pulsed corona discharge plasma	High-voltage electrode: 19 stainless-steel hypodermic hollow needles Ground electrode: wire netting Distance between adjacent needles: 12.5 mm Distance between electrodes: 16 mm Pulse frequency: 50 Hz Pulsed discharge voltage: 18 kV Pulse-forming capacitance: 200 pF	PNP degradation: 86% PCP degradation: 94.1% Energy yield: 18.3% Degradation efficiency decreases with increasing initial pollutant concentration.	[177]
*p*-nitrophenol (PNP)	Dielectric barrier discharge	Voltage: 38.2 kV High-voltage electrode: stainless steel Dielectric barrier: quartz glass Working gas: air	PNP degradation: 63.2% The treatment time, applied discharge voltage, and soil pH value have a positive effect on the degradation efficiency. Airflow is harmful to the decomposition process. The ozone plays an important role as an active species in gas form.	[173]
Glyphosate	Dielectric barrier discharge	Optimal discharge voltage: 28 kV Power-frequency discharge: 50 Hz Distance between probe and ground electrode: 5 mm	Glyphosate degradation: 93.9% Energy yield: 0.47 g kWh^−1^ Increasing the discharge voltage and decreasing the organic matter content of the soil facilitate glyphosate degradation.	[172]
Atrazine	Dielectric barrier discharge	High-voltage electrode: stainless-steel disc Dielectric barrier: quartz Ground electrode: stainless-steel grid Voltage power supply: 34.2–44.8 kV Working gas: dry compressed air	Degradation efficiency: 86.9% and 98.1% for initial concentrations of 100 and 10 mg/kg, respectively. A low soil moisture content (5–10%) enhances atrazine degradation. Atrazine mineralization: 65.5% Main oxidizing agents: OH·, H_2_O_2_, or O_3_	[49]
Trifluralin	Dielectric barrier discharge	High voltage: 20 kV High-voltage and grounded electrode: stainless steel Dielectric barrier: quartz tube Gas flow rate: 0.075 L/min Working gas: compressed air	The degradation of trifluralin is feasible, even in thicker soil. The degradation efficiency decreases by 30% with increasing soil moisture. The energy efficiency is up to three orders of magnitude.	[174]

**Table 10:** Summary of studies on the degradation of pesticide residues in food using cold plasma technology.

Pesticide	Food Product	Plasma System	Plasma Configuration	Key Findings	Reference
Dichlorvos and omethoate	Maize	Radiofrequency (RF) discharge	Working gas: oxygen Power supply: 500 W, 13.56 MHz Reaction chamber: cylindrical Pyrex glass tube	This treatment was significantly effective in the degradation of original DDVP and omethoate. The degradation efficiency mainly depends on the related operating parameters and chemical structures of the pesticides. DDVP and omethoate molecules are degraded into less toxic compounds.	[179]
Azoxystrobin, cyprodinil, fludioxonil, and pyriproxyfen	Strawberries	Dielectric barrier discharge	High-voltage electrode: Perspex Ground electrode: polypropylene Package container: polyethylene terephthalate (PET) High-voltage output: 0–120 kV Frequency: 50 Hz Working gas: atmospheric air	Maximum decrease (5 min, 80 kV) 69% of azoxystrobin 45% of cyprodinil 71% of fludioxonil 46% of pyriproxyfen Plasma treatment is a means of ensuring chemical food safety and microbicidal effects.	[163]
Diazinon	Cucumber	Dielectric barrier discharge	Working gas: air Upper electrode: copper Dielectric barrier: quartz Second electrode: stainless-steel mesh Pulsed high voltage: 0–14 kV Frequency: 6 kHz	Degradation efficiency depends on the plasma treatment time, discharge power, and pesticide concentration. The produced organophosphate pesticides are harmless and less hazardous compounds.	[183]
Diazinon and chlorpyrifos	Apples and cucumbers	Dielectric barrier discharge	Frequency: 13 kHz Distance between electrodes: 7 mm Exposure time: 10 min Voltage: 13 kV	Cold plasma considerably reduces the amount of pesticide residues without leaving any trace of harmful or toxic substances. No undesirable effects on the color or texture of the samples were noted. The efficiency increases with a higher voltage and a longer exposure time.	[185]
Boscalid and Imidacloprid	Blueberry	Dielectric barrier discharge	Electrodes: aluminum plate Package container: polyethylene terephthalate (PET) Dielectric barrier: PET Working gas: atmospheric air High voltage output: 80 kV Treatment time: 5 min	Degradation efficiency: 80.18% for boscalid 75.62% for imidacloprid The total phenol and flavonoid contents of blueberries increase significantly after plasma treatment. There is no significant effect on physical parameters.	[186]
Omethoate and dichlorvos	Goji (*Lycium barbarum*)	Gas-phase surface discharge (GPSD)	GPSD setup comprises tungsten wires (150 µm), hollow-core quartz fibers, and a bipolar high AC voltage Plasma exposure time: 30 min Discharge voltage: 10 kV	The degradation depends significantly on the applied voltage and the plasma exposure time. Omethoate degradation: 99.55% Dichlorvos degradation: 96.83% Omethoate and DDVP molecules can be completely degraded into nontoxic species without compromising the quality of *Lycium barbarum*	[193]
Chlorpyrifos and carbaryl	Maize	Dielectric barrier	Two aluminum electrodes Two glass dielectric barriers Distance between electrodes: 6 mm Working gas: argon	Chlorpyrifos degradation: 91.5% Carbaryl degradation: 73.1% This treatment improved the hydrophilicity of the treated maize. No significant change in the vitamin B2 content of maize was noted. A significant increase in the acid value and a decrease in the moisture and starch contents was observed.	[194]
Chlorpyrifos and carbaryl	Grapes and strawberries	Pin-to-plate atmospheric plasma discharge	High-voltage electrode: pin array Ground electrode: flat plate Distance between the pins and the ground electrode: 7 cm A resonant frequency: 55.51 kHz A discharge voltage: 32 kV Input power: 5.66 W	Chlorpyrifos degradation: 79% on grapes and 69% on strawberries Carbaryl degradation: 86% on grapes and 73% on strawberries Important factors for pesticide dissipation include nitrates, nitrites, and hydrogen peroxide. No significant changes in the key physical attributes (color and firmness) were noted. Slight changes in the ascorbic acid levels were observed.	[191]
Chlorothalonil (CTL) and thiram (THM)	Tomato (*Solanum lycopersicum*) fruit	PAW and plasma-activated buffer solution (PABS)	Working gas: atmospheric air	CTL degradation: 85.3% with PAW and 74.2% with PABS THM degradation: 79.47% in PAW and 72.21% in PABS Increasing the activation time results in a significant reduction in the amount of fungicide residues. Oxidation–reduction potential (ORP) and electrical conductivity (EC) improve significantly after plasma treatment, while the pH value decreases with the activation time. No notable negative impact was observed on tomatoes.	[195]
Chlorpyrifos and carbaryl	Corn	Dielectric barrier discharge	Two aluminum electrodes Dielectric barrier: glass Working gas: air Gap between two electrodes: 6 mm Plasma treatment time: 60 s Air flow rate: 1000 mL/min Power: 20 W Frequency: 1200 Hz	Chlorpyrifos degradation: 86.2% Carbaryl degradation: 66.6% A remarkable decrease in the moisture and starch contents was noted. The vitamin B2 content of treated corn does not show a significant difference from that of untreated corn.	[190]
Chlorpyrifos and cypermethrin	Mango	Gliding arc discharge	Plasma treatment time: 5 min Working gas: argon Ar flow rate: 5 L/min Transformer power: 600 W	Chlorpyrifos degradation: 74.0% Cypermethrin degradation: 62.9% A significant decrease in titratable acidity and total phenolic content was noted. There was an increases in carotenoid content. Total soluble solid, color, and texture parameters were not significantly different.	[47]
Cypermethrin	Tangerine	Pinhole plasma jet	DC power supply: 15 kV. Acrylic container: 410 × 290 × 90 mm Electric power: 125 W Working gas: air Air flow rate: 15 L/min. Discharge time: 60 min	Cypermethrin reduction: 0.75 ppm Tangerine exhibits longer shelf-life after treatment. No significant differences were noted in appearance, acid flavor, sweetness, and smell.	[196]
Phoxim	Grapes	Plasma jet	Plasma discharge time: 30 min Treatment time: 10 min Working gas: air Power supply: alternating current Frequency: 20 kHz Air flow rate: 5 L/min Plasma jet under water: 2 cm	Phoxim degradation: 73.60%. Acidic PAW environment: pH < 3. Oxidation capacity: >500 mV. Treatment does not significantly affect the qualities of grapes, including color, firmness, sugar content, vitamin C, and SOD.	[139]
Chlorpyrifos	Tomato	Dielectric barrier discharge	Treatment time: 15 min Air flow rate: 10 L/h. Initial concentration: 0.8 mg/kg. Input voltage: 200 V. Working gas: air.	Maximum reduction in chlorpyrifos: 51.97%. The total color index was increased significantly. The texture of the tomato was unaffected after PAW treatment.	[197]
Chlorpyrifos	Tomato	Dielectric barrier discharge	Electrodes: aluminum Glass dielectric: 2 mm Frequency: 50 Hz Distance between electrodes: 5 cm Plasma exposure time: 6 min Plasma reactor size: 350 × 350 × 350 cm	Maximum reduction of chlorpyrifos: 89.19% Initial concentration: 0.6 ppm The color index (TI) was significantly enhanced. Firmness, bio yield point, carotenoids, and total phenolic contents were decreased considerably.	[180]
Malathion and chlorpyrifos	Lettuce	Dielectric barrier discharge	Frequency input: 50 Hz High voltage output: 0–130 kV Distance between electrodes: 40 mm Treatment time: 180 s	Malathion degradation: 64.6% Chlorpyrifos degradation: 62.7% No significant damage was noted in regards to color and chlorophyll content. Ascorbic acid decreased significantly during long-term treatment.	[184]
Chlorothalonil fungicide	Tomato	Plasma-activated water (PAW) and plasma-activated buffer solution (PABS)	Power output: 600–1000 W Operating voltage: 2–7 kV Working gas: dry air Air flow rate: 20 L/min Distance between nozzle exit and liquid surface: 30 mm. Treatment time: 15 min.	Chlorothalonil reduction: 89.28% (PAW_10_-U) Chlorothalonil reduction 80.23% (PABS_10_-U) Degradation products: 2,4,5-trichloroisophthalonitrile, 2,4-dichloroisophthalonitrile, 4-chloroisophthalonitrile, isophthalonitrile and phenylacetonitrile. No negative effects were observed regarding tomato quality.	[198]
Carbendazim and chlorpyrifos	Chili	Pinhole plasma jet-activated water	Working gas: argon and 2% oxygen Anode electrode: tungsten Cathode electrode: aluminum blade Gas flow rate: 10 L/min	The efficiency of pesticide degradation is higher on the chili surface than in the solution. Carbendazim and chlorpyrifos degradation rates of 57% and 54% were noted in the solution, respectively. Carbendazim and chlorpyrifos degradation rates of 80% and 65% were observed on the chili surface.	[192]

## Data Availability

Not applicable.
